# Mechanical Mechanism of Ion and Water Molecular Transport through Angstrom-Scale Graphene Derivatives Channels: From Atomic Model to Solid-Liquid Interaction

**DOI:** 10.3390/ijms241210001

**Published:** 2023-06-11

**Authors:** Lei Fan

**Affiliations:** School of Civil Engineering and Architecture, Zhejiang University of Science & Technology, Hangzhou 310023, China; fanleigl@foxmail.com or fanleigl@shu.edu.cn

**Keywords:** graphene derivatives, angstrom-scale channels, ion and water molecular transport, atomic model, mechanical mechanism in solid-liquid interaction

## Abstract

Ion and water transport at the Angstrom/Nano scale has always been one of the focuses of experimental and theoretical research. In particular, the surface properties of the angstrom channel and the solid-liquid interface interaction will play a decisive role in ion and water transport when the channel size is small to molecular or angstrom level. In this paper, the chemical structure and theoretical model of graphene oxide (GO) are reviewed. Moreover, the mechanical mechanism of water molecules and ions transport through the angstrom channel of GO are discussed, including the mechanism of intermolecular force at a solid/liquid/ion interface, the charge asymmetry effect and the dehydration effect. Angstrom channels, which are precisely constructed by two-dimensional (2D) materials such as GO, provide a new platform and idea for angstrom-scale transport. It provides an important reference for the understanding and cognition of fluid transport mechanism at angstrom-scale and its application in filtration, screening, seawater desalination, gas separation and so on.

## 1. Introduction

When people see civil engineering, environmental engineering and biomedical engineering side by side, they could be puzzled. These disciplines are quite different. Why are they discussed together? Miraculously, through the study of ion and water molecule transport in nano-channels of GO, we can make them “marry”, which leads to research crossover and disciplinary exploration.

In recent years, the idea of using GO channels under various conditions and for a variety of applications has engaged the minds of many researchers [1]. Nanopores/channels are ubiquitous in biological systems. Furthermore, the transport of ion and water molecules in the retina, nerves, muscles and other living systems plays a key role in life activities [2]. Inspired by biological GO nano-channels in cell membranes, artificial nanopores/nano-channels have been successfully constructed. It is used to transport ions and water molecules directionally by adjusting the interface interaction [3]. Studying the basic principle of ion and water molecule transport in GO nanopores/nano-channels will help to further improve the performance of different artificial materials. Based on these principles, GO nanopore/nano-channels have a wide range of applications, such as oil-water separation, seawater desalination, controlled drug delivery, salt difference power generation, pressure power generation, concrete lifetime, DNA sequencing, environmental monitoring and so on [4,5].

At the nanometer scale, the interface effect and intermolecular force are prominent, and the free movement of water molecules is limited. Therefore, the GO nano-channels show the characteristics of layering and ordering [6].

It is worth noting that the variable arrangement of oxygen-containing functional groups, the complex stoichiometric ratio, the various spatial configurations and the various preparation methods in GO will lead to the uncertainty of the interface structure and interface interaction of GO and the transport behavior of ion and water molecule in GO nano-channels.

In this paper, the chemical structure and molecular model of GO are discussed first, and then, the preparation of nano-channels with adjustable structure and shape by using the uniqueness of GO structure is introduced. Next, the transport characteristics of ions and water molecules in GO nano-channels and the mechanical mechanism of nanofluids in the nano-channels are discussed. These basic studies have found many unexpected behaviors and “strange” characteristics of substances at the nanometer scale. It is expected to solve the “difficult problems” in the application fields of civil engineering, biomedical engineering and environmental engineering, especially the challenges in the water-energy field.

## 2. Structure and Theory of Graphene Oxide

The precursor of GO is graphite oxide, which is formed by the intercalation and oxidation of graphite particles from the internal surface to the internal under the action of strong oxidants (such as KClO_3_, KMnO_4_, etc.). The reaction mechanism is still unclear. As a non-stoichiometric compound, GO has a large number of oxygen-containing FGs on its surface, but there is no definite stoichiometric ratio and arrangement [7]. Compared with graphene whose structure is almost perfect and definite, the structure of GO is very complicated.

At present, many structural models have been put forward based on the structure and properties of GO. The most famous GO models are the Lerf–Klinowski model (A. Lerf and J. Klinowski proposed), the Dynamic structure model (A. M. Dimiev proposed) and the Two-component structure model (J. P. Rourke proposed). 

### 2.1. Lerf–Klinowski Model

**Figure 1:**
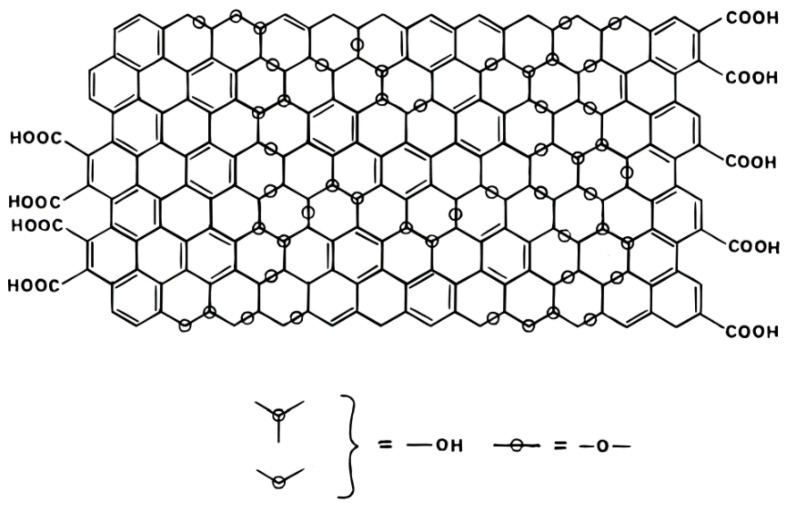
The LK structure model of GO proposed by A. Lerf and J. Klinowski et al. [11].

GO has many kinds of oxygen-containing FGs, including epoxy, carboxyl, carbonyl, hydroxyl groups and so on [8]. However, there is no definite element ratio and chemical formula, and there is also a lack of powerful and accurate characterization technology [9]. Therefore, the controversial focus of GO structure is the distribution and arrangement of FGs [10]. A. Lerf and J. Klinowski proposed the Lerf–Klinowski (LK) model according to ^13^C and ^1^H nuclear magnetic resonance (NMR) data of GO and its specific derivatives. Their findings indicate that two kinds of atomic structure regions exist on GO nanosheets [11]. One is the oxidation zone with a large number of FGs, in which the carbon atoms are mainly sp^3^ hybrid. The other is the graphite lattice region (no FGs) mainly composed of sp^2^ hybrid carbon atoms [11]. They also showed that the hydroxyl and epoxy groups are mainly distributed in the oxidation zone, while carboxyl groups are distributed at the edge of the GO sheet [12]. The LK structure model of GO is shown in Figure 1.

So far, different characterization techniques, such as solid-state NMR, X-ray photoelectron spectroscopy (XPS) or Raman spectroscopy, have been used to prove the accuracy of the GO surface theoretical model. D. A. Sokolov et al. [13] observed the light emission and absorption of GO in situ during the laser reduction of GO. Their results confirmed the spatial heterogeneity of light emission and absorption in a single GO. A. Tararan et al. [14] used Electron energy loss Spectroscopy (EELS) to investigate the oxygen content in specific areas of GO. Their findings show that the oxygen content in some oxidation areas of GO was as high as 50%. More importantly, the groups in these regions are all hydroxyl groups, rather than a mixture of epoxy groups and hydroxyl groups. The result is inconsistent with the LK model. The LK model alone cannot fully explain the chemical properties of GO and the LK model cannot explain the strong acidity of GO.

### 2.2. Dynamic Structure Model

**Figure 2:**
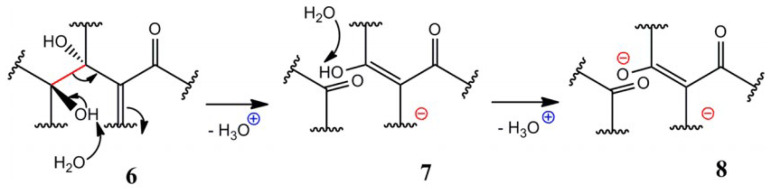
A possible situation of a reaction with GO and H_2_O based on the DSM model (Proposed A. M. Dimiev). The C-C bond (structure **6**) between the two hydroxyl groups was broken under the attack of H_2_O and ketone and an enol (**7**) was generated, leaving H_3_O^+^. The newly formed enol combines with the carbonyl group to form intercalated carboxylic acid, which could be hydrolyzed to **8** [15,16].

Based on the contradiction between GO’s strong acidity and structure, A. M. Dimiev et al. improved the LK model and proposed a Dynamic Structure Model (DSM) [15]. In the DSM model, Dimiev et al. believe that acidity is the key to understanding GO structure [15]. In another work, the content of acidic sites in GO was analyzed by a potentiometric titration experiment [16]. According to ^13^CSSNMR data, it was speculated that there were acidic groups in GO. It was also maintained that the acidity of GO mainly comes from the structure of alkene-like alkyd in GO and the hydrolysis of residual covalent sulfate, rather than the carboxyl group of GO [16], as shown in Figure 2. Among them, one possible method is to form an intercalated carboxylic acid. The C-C bond (structure **6**) between the two hydroxyl groups was broken under the attack of H_2_O, and ketone and an enol (**7**) were generated, leaving H_3_O^+^. The newly formed enol combines with the carbonyl group to form intercalated carboxylic acid, which could be hydrolyzed to **8**. In addition, A. M. Dimiev and T. Szabó et al. [16,17] proposed a DSM model considering nanohole and defects. The model also confirmed that the cleavage of the C-C bond in GO formed ketone and enol.

### 2.3. Two-Component Structure Model

**Figure 3:**
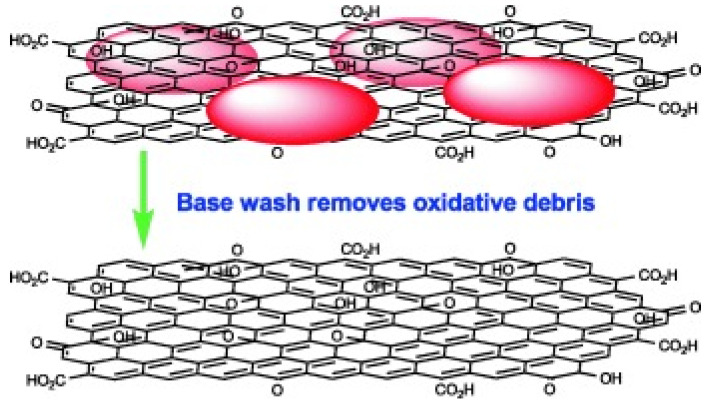
The two-component structure model proposed by J. P. Rourke et al. [18].

The DSM model explains the molecular structure and composition of GO from the aspects of electricity, chemistry and electrochemistry. J. P. Rourke et al. [18] proposed the Two-component structure (TCS) model based on the LK and DSM models, combined with research on oxidation and acidification of carbon materials such as CNTs [18]. The TCS models are shown in Figure 3. In the TCS model, they considered that the structure of GO should be divided into two parts, including Base washed GO (BW-GO) and Oxidation debris (OD) [18,19]. The TCS model is proposed mainly because OD will be produced in the oxidation preparation process of GO [19]. Moreover, J. P. Rourke found an important conclusion by heating and refluxing GO in an alkaline solution [18,19,20]. They also maintained that GO is not a reduction reaction but a cleaning process in an alkaline environment. In neutral and acidic environments, OD is strongly adsorbed on the surface of BW-GO. However, OD could be separated from BW-GO in an alkaline environment [20].

Although the TCS model has been partially recognized, there are also voices of doubt [21]. A. M. Dimiev, the proponent of DSM, questioned J. P. Rourke’s TCS model from four aspects [21]: (a) Obversion of OD, (b) The change of GO after alkali treatment GO = BW-GO + OD?, (c) Separation of OD, and (d) Origin of OD. Among them, whether OD has been identified by microscopic observation (that is, confirmed to exist) is the key to A. M. Dimiev’s questioning of the TCS model [22]. A. Tararan et al. [14] used low voltage, low dose and liquid nitrogen cooling in the TEM observation of GO to avoid damage to the sample structure. Their observation results did not support the existence of OD. In addition, S. H. Dave observed a clear GO atomic structure image at 700 °C [23]. However, GO’s adsorption of pollutants blurs the image. Therefore, the existence of OD has not been proved.

Whether GO is a substance composed of one component or two components, the relevant controversy will continue. However, it is worth noting that the TCS model no longer only focuses on the atomic/molecular structure of GO, but focuses on the layer and macromolecule of GO.

## 3. Nano/Angstrom Channel of Graphene Oxide

A. K. Geim et al. [24] proposed the concept of molecular screening with GO film for the first time. They prepared sub-micron GO film by the spin coating method, and tested the permeability of various liquids and gases to GO film. GO membrane showed a complete barrier to some organic solvents and gases (Ar, He, etc.), but almost had no obstacle to the permeation of water vapor [24]. The reason is the special structure of GO lamellae, which is composed of two parts: original graphene lamellae and it is surface rich in FGs, such as hydroxyl, carboxyl, carbonyl, epoxy and so on.

These hydrophilic groups help to attract water into the interlayer channels inside the GO film, and the friction-free super lubrication of the original graphene area in the channels makes the water molecules flow rapidly between the layers [24]. In another work, it was also confirmed that sub-micron GO films can effectively filter out salt ions with a hydration ion radius larger than 4.5 Å in solution [25].

**Figure 4:**
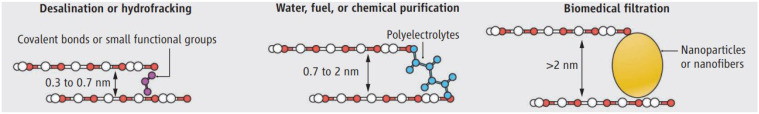
The separation capability of the GO membrane is tunable by adjusting the nanochannel size [29].

GO is mostly composed of multi-layer structures, and so these layered structures often contain such microstructures as lamellar defects, lamellar cracks and capillary channels between lamellar layers [26]. Therefore, by utilizing the high regulation of nano-channels between GO layers and adjusting the relevant parameters of GO films, the directional screening from organic macromolecules to small ions can be realized under different conditions [27,28]. If the chain polymer electrolyte is inserted between layers, the channels can be adjusted to 0.7–2 nm. It can be used to obtain clean water, fuel and chemical purification. If nanoparticles or nanofibers are inserted between layers, the channels can be adjusted to more than 2 nm. It can be used for biomedical treatment such as hemodialysis (see Figure 4) [29]. Moreover, Quantum filtering using entropy filters made of carbon-based materials (graphene, carbon nanotubes, graphene oxide, etc.) is particularly interesting, because these carbon-based materials can be obtained in various forms and their microstructures can also be controlled manually [30]. J. Niechcial et al. [31,32] proposed an effective separation technique for He4/He3 through an entropy filter composed of carbon nanotubes based on the principle of atomic scale channels.

### 3.1. Transport Properties of Ions and Water Molecules in GO Nano/Angstrom Channels

GO is rich in epoxy groups and hydroxyl groups on the lamellar plane, while the edges are mainly carbonyl groups and carboxyl groups [33]. At the same time, it can also be seen that a variety of oxygen-containing FGs (hydroxyl, epoxy, carbonyl, carboxyl and so on) are embedded on the surface and edge of the 2D lattice of graphene [34,35]. Therefore, a unique structure isolated from the sp^3^ C-O matrix by numerous sp^2^-hybridized carbon atom clusters is generated. For GO separation membranes, the formation of water molecular permeation channels is mainly due to the stacking of sp^2^ regions of GO sheets. However, the oxidation area in the middle and edge of GO lamella tends to cluster due to hydrogen bonding with water molecules. It is not conducive to the penetration of water molecules [36]. According to the screening theory, the separation membrane can allow particles smaller than its pore size to penetrate the membrane, while components larger than its pore size can be intercepted [37]. For example, for GO films with a layer spacing of 9 nm, larger organic solute molecules (>1 nm) can be completely blocked by GO films, while for hydrated ions with a smaller radius (such as Na^+^, the hydration radius is about 0.3–0.7 nm), they can permeate through the films under the action of the nano-capillary force of GO films. Therefore, it has been suggested that the precise screening of particles with different sizes can be achieved by adjusting the interlayer spacing of GO films [38,39]. On the one hand, the interlayer spacing of GO films can be increased by inserting large-sized nanoparticles between GO sheets. When the interlayer spacing of GO films is larger than 2 nm, a precise sieving system can be achieved in applications of biomedical such as artificial kidney and hemodialysis. On the other hand, the interlayer spacing can also be reduced by decreasing the distribution of oxygen-containing FGs.

Considering the wettability of the channel wall and the influence of FGs, Y. Zhang et al. have studied the effect of confined water on the nanochannel wall [40]. When the spacing of nanochannel layers is constrained within 1.4 nm, it can only exist stably at a few specific spacings. The discontinuous quantization of stable layer spacing can be attributed to the layered structure formed by water molecules in the confined space [40].

**Figure 5:**
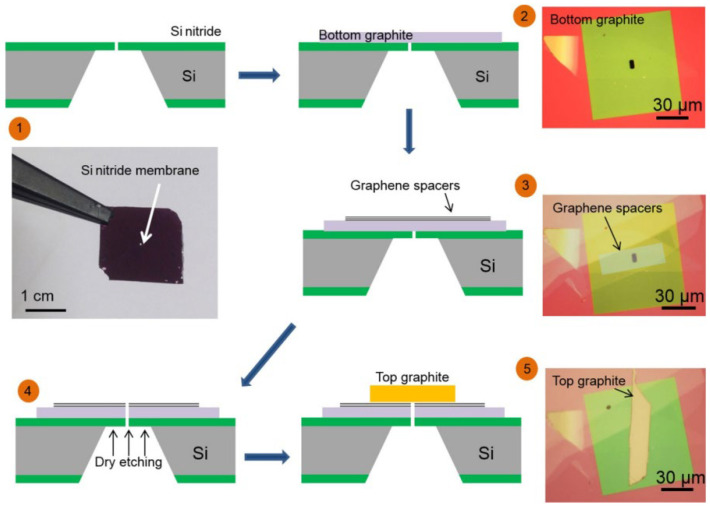
Schematic diagram of the three-dimensional structure of nano-channels with atomic level accuracy obtained by using five steps: (1) A micrometer-scale hole is prepared in a silicon nitride membrane. (2) The graphite at the bottom is transferred to cover the opening. (3) The graphene spacer array was transferred to the top. (4) By dry etching, the holes extend into the graphite-graphene laminate. (5) The graphite crystal at the top is transferred to cover the resulting pores. The width W of the channel is 130 nm, the length L is between 1 and 1~10 μm, and the thickness H can be controlled according to the number of graphene layers (N) (the interlayer spacing of graphene in this study is a=3.4 Å) [41].

B. Radha et al. [41] studied the transport behavior of water molecules in channels with different sizes. By using micromachining technology, the nano-channels with adjustable atomic accuracy and size were prepared, as shown in Figure 5. Their results indicated that the water transport is related to the effective length L and thickness H inside the channel. The smaller L is, the more favorable it is for water transport. However, the transport speed of water is not big when H is small or large. The transport flow Q of water per micron of each nano-channel reaches the maximum value (close to $2\times 10^{-10}\;g\cdot s^{-1}$) when N=4 (there are four layers of graphene in the spacer layer). In addition, when the water nano-channel is transported by capillary effect, the maximum speed can reach 1 m/s [41]. This is due to the internal separation pressure of 1000 bars (1 bar = 1 atmosphere). 

**Figure 6:**
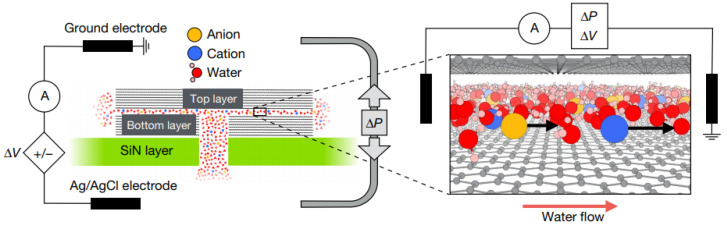
Experimental setup for pressure- and voltage-driven current. Each device had *N* = 200 channels of height h=6.8 Å, width w=130 nm. The graphite or h-BN as the top layer and the bottom layer (channel wall material) [43].

According to the classical Knudsen diffusion theory, when gas molecules are transported inside the space far less than their average free path, the collision opportunity between molecules and walls is greater than that between molecules. Therefore, the resistance of material diffusion along the hole mainly depends on the collision between molecules and walls, that is, there is a very obvious diffuse scattering phenomenon, which will hinder the transport of molecules. A. Keerthi and A. K. Geim et al. [42] discovered this molecular ballistic transport with a large number of specular reflections by adjusting the wall materials and sizes of nano-scale channels. It is proved that molecules are not necessarily transported by the Knudsen diffusion mechanism in space far below their average free path. When graphene or h-BN with very high surface roughness is used as the channel wall material, the He atom has a very high transport flow in the channel with a height of 1.7 nm, which is much higher than the calculated value of Knudsen’s diffusion theory formula (2 orders of magnitude). However, in the channel with the same height, when MoS_2_ with low surface flatness is used as the channel wall material, its transmission flow rate is low and close to the calculated value of the Knudsen diffusion theory formula [42]. In 2019, T. Mouterde and A. K. Geim et al. [43] first prepared a test device with double-layer graphene as a spacer layer (each device had *N* = 200 channels of height h=6.8 Å, width w=130 nm) and graphite or h-BN as the top layer and bottom layer (channel wall material) according to the process and method in Figure 6. Then, a KCl-containing solution tank and a current testing device (Ag/AgCl electrode) of the whole channel are connected at both sides of the channel. When the KCl solution is transported in the Emmy channel, the applied voltage and pressure are changed. Next, the influence of the external disturbance on the ion transport behavior is studied through the change of system current. Under the condition of no applied voltage (ΔV=0), the transport behavior of molecules/ions shows that the path current has a good linear relationship with ΔP. The result confirmed that the current of the system comes from the hydrodynamic transport of ions. Although there is little correlation between the path current and the concentration of the KCl solution, the absolute value of the electroosmotic mobility finally calculated is very large, which can reach the magnitude of 10^−7^ (m^2^V^−1^s^−1^) ($u_{K+}=7.6\times 10^{-8}\;m^{2}V^{-1}s^{-1}$ under macroscopic conditions). In addition, different from the results of graphite (alkene) 2D Emmy channel, the current and electroosmotic mobility in 2D h-BN channel have a good linear relationship with KCl solution concentration and voltage, while the same with graphene Emmy channel is that the current and pressure have a linear relationship, and the values of current and electroosmotic mobility will increase rapidly at the lower voltage. An important conclusion can be obtained that the ion migration in the pressure-driven 2D Angstrom channel is highly reactive to the voltage [43].

Professor Andre Geim’s research group’s three Nature articles show that the fluid transport dynamics in the nanometer or even Angstrom-scale confined space will be different from that in the macroscopic state when the material is transported in a single molecule state. With the gradual maturity of the preparation technology of nano and even sub-nano devices, more and more people pay attention to the relationship between the transport behavior of materials on the 2D ultimate scale, the physical and chemical properties of fluids and materials and the size of channel structures. The transport mechanism of nano-channels has important guiding significance for the development and preparation of such subversive technologies and devices as clean energy, sensing, molecular/ion separation and biomedicine at the ultimate scale.

### 3.2. Hydratedion: Charge Asymmetry Effect and Dehydration Effect

Because the migration process of ions cannot be separated from the solution and exist alone, the hydration state of ions in an aqueous solution should be considered when ions migrate in GO nano-channels [44]. More importantly, the charged ions will combine with a certain number of surrounding water molecules to form hydrated ions [44]. It is worth noting that the size of the hydrated ion will be larger than that of the ion itself. Therefore, the pore size or layer spacing corresponding to the hydrated size of the ion is often selected when measuring the screening effect. In addition, different ions have different charges, which results in different binding capacities between different ions and water molecules, thus forming different ion hydration structures [45].

**Figure 7:**
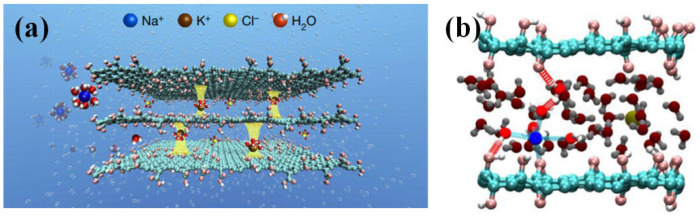
Ionic “binding” GO film for ion screening and seawater desalination. (**a**) A schematic diagram of how K^+^ ions in GO films determine and fix the interlayer spacing, thus rejecting other cations and allowing pure water to permeate. Yellow pillars between the GO sheets describe the fixation of the interlayer spacing by hydrated K^+^. (**b**) Theoretical computations for cations between two GO sheets [46].

**Figure 8:**
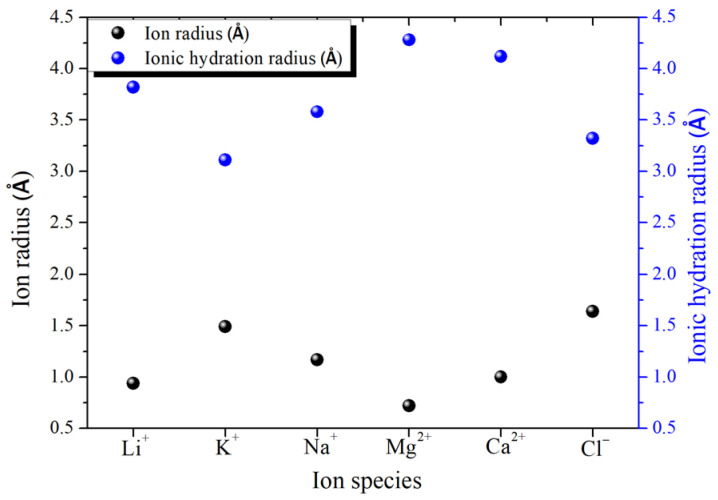
The ion radius and ionic hydration radius of common ions [24,25].

For low-valence ions such as sodium and potassium plasma, they can only maintain a slightly tight binding force with hydrated water molecules in the surrounding layer, as shown in Figure 7 [45]. Furthermore, because of their weak attraction, the bound water molecules are easily affected by the surrounding environment during migration. Some divalent ions, such as calcium and magnesium plasma, have a stronger binding force than monovalent ions, so divalent ions can attract some water molecules in addition to one layer of hydrated water molecules, forming a two-layer hydrated structure [46]. Higher valence ions, such as aluminum ions, can even form relatively stable coordination bonds with water molecules, and this super-strong binding ability exists in the form of complex ions [47]. Many studies [48,49] have pointed out that the grading screening effect of different ions can be achieved by designing the corresponding nanostructure size in graphene-derived materials according to the hydration radius of the selected ions. The ion radius and ionic hydration radius of common ions are shown in Figure 8.

Next, we turn our attention to the relationship between ionic hydration radius and nano-channels sizes. A previous study [50] has shown that when hydrated ions try to pass through a nano-channel with a height less than $D_{h}$, the ions may be completely rejected due to the size exclusion. In other words, it will cause a larger energy barrier and may change the selectivity of some ions. Nano-channels smaller than $D_{h}$ hinder the rapid diffusion of ions (about 10^−11^ to 10^−13^ m^2^/s), while the typical angstrom size and measured diffusion coefficient of hydrated ions are equivalent to or even slower than that of bulk water (~1.9 × 10^−9^ m^2^/s) [51]. Y. H. Xue et al. reported an atomic-level ion transistor based on a gated graphene channel (height ≈ 3 nm). The average surface potential of the graphene layer is controlled by the applying electric gate, thus changing the energy barrier of the ion insertion channel. The graphene nano-channels have ultra-fast and selective ion transport, which are two orders of magnitude faster than ion diffusion in water (2.0 × 10^−7^ m^2^/s) [51]. A. K. Geim and K. Gopinadhan jointly studied the transport behavior of ions in the ultra-strong confined Ami-channel composed of graphene, h-BN and MoS_2_ [52]. It was found that the space effect led to little surface charge on the inner wall of the 2D channels. When the diameter of hydrated ions was larger than the size of the 2D channel, they could still be transmitted and penetrated at a lower speed. At the same time, this effect has obvious asymmetry between anions and cations with the same diameter [52]. Y. Z. Yu et al. [53] employed molecular dynamics simulation to study the charge asymmetry effect of cation and anion mobility in nano-channels. They showed that the mobility of potassium ions and chloride ions is consistent in bulk solution. However, the mobility of chloride ions is smaller than that of potassium ions when they are transported in the Angstrom channel. The difference in fluidity is due to the subtle variances of their respective hydration layer structures, and the differences in their relatively stable positions in the channel after balancing with double-layer water in the confined space [53]. In addition, ion mobility, the effect of charge asymmetry on hydration-free energy is also reported [54].

A previous study [55] has shown that the structure of the ion hydrate layer will rearrange and integrate (including the deformation and partial dehydration of the hydrate layer) when entering the channel, to adapt to the strongly confined space. When the channel size reaches the material scale limit, i.e., the Angstrom scale, the dehydration effect at the channel port seriously affects the conductivity of ions, and even completely prevents ions from entering the nano-channels [55]. Y. Z. Yu et al. [56] studied the effect of size exclusion on ion transport into 2D Angstrom-scale channels and obtained the molecular details of the dehydration process. Their findings indicated that the barrier size strongly depends on the hydrate diameter and channel size under the strong confinement of nano-channels [56]. The energy barrier at the channel port is an important intrinsic factor that hinders the ionic conductivity in the 2D Angstrom-scale channel.

The whole process of hydrated ions passing through nano-channels is quite complex and has not been described clearly yet, especially when the channel size is close to the size of ions and water molecules.

### 3.3. Mechanical Problems of Nano-Fluidics in Solid/Liquid Interface

Nano-fluid studies the transport behavior of substances in nano-scale channels. Although solid-state physics has studied nano-fluidics for a long time, the nano-fluidics devices required for systematic research on nano-fluidics are a major bottleneck hindering the development of the nano-devices field [57]. Due to the lack of in-depth understanding of mass transfer mechanisms and control methods, it is difficult to quantitatively describe the limited mass transfer. The problem of limited mass transfer restricts the application of related materials and structures [58]. Therefore, scientists’ experimental research on the transport behavior of molecules, ions and other substances in nano-channels has only been 15 years. In a confined mass transfer, traditional hydrodynamic models generally have serious deviations and cannot accurately predict the flow flux [59,60]. In addition, the prominent wall effect of 2D materials has a significant impact on fluid flow, which makes the controlled factors of confined mass transfer more specific [61]. All these problems put forward further requirements for the development of the nano-hydrodynamics theory. In recent years, with the emergence of a large number of new nano-materials and fine processing technologies for preparing nano-channels, research progress in the field of nano-fluidics has made a huge leap. L. Bocquet [62] pointed out in an article by Nature Materials that the age of nano-fluidics has come!

The measurement results of earlier studies showed that there was little friction when water passed through the nanotubes (the speed of liquid flow per second was only one billionth of a liter). This can be attributed to the fact that the walls of carbon nanotubes are completely smooth. The lack of surface roughness of nano-channels reduces the resistance through water molecules. In 2016, L. Bocquet and D. Stein [63] studied the transport behavior of water molecules in nano-scale channels. They showed that radiation-dependent flow slips in carbon nanotubes can make water molecules flow at high speed without friction. However, boron nitride nanotubes, which have the same crystal type as carbon nanotubes, but different electronic structures, have no such phenomenon. Hence, the transport behavior of materials in nano-channels is closely related to the properties of the solid-fluid interface at the atomic scale [63]. An important conclusion can be reached; the friction depends on the radius of the nano-channels. Confusingly, the friction effect increases on the larger nano-channels, which does not make any sense, because the larger nano-channels should be as smooth as the smaller ones. These strange phenomena have caused controversy in the physical field and become a key knowledge gap in the research of nano-scale flow [63]. In 2022, a new theoretical research report published in Nature said that N. Kavokine et al. had finally found the answer: “Quantum friction” [64]. The electrons on the graphene wall will move with the passing water molecules. However, electrons tend to lag behind slightly, thus slowing down the speed of molecules. This effect is called electronic or quantum friction, which was previously only considered as a factor in the interaction between two solids or a single particle and a solid [64]. However, when it comes to liquid, the situation is more complicated, because there are many molecules interacting in liquid. Furthermore, electrons and water molecules vibrate due to their thermal energy. If they happen to vibrate at the same frequency, they will have a resonance effect and increase quantum friction. This resonance effect is greatest for nanotubes with well-aligned layers because the movement of electrons between layers is synchronized with the movement of water molecules [64]. The latest development can foresee not only the basic discovery of the final scale fluid transportation but also the disruptive technology between water and energy.

## 4. Application of GO Nano-Channels

### 4.1. Application of GO Nano-Channels in Civil Engineering

Calcium silicate hydrate (C-S-H) is one of the main hydration products of concrete materials, accounting for about 60–70% of the total volume of hydration products. It is one of the main sources of concrete strength and has a significant impact on the durability of concrete [65]. C-S-H gel has a layered structure, and there are a lot of micropores between layers, including small gel pores (5~100 Å) and capillary pores (>100 Å) [66].

**Figure 9:**
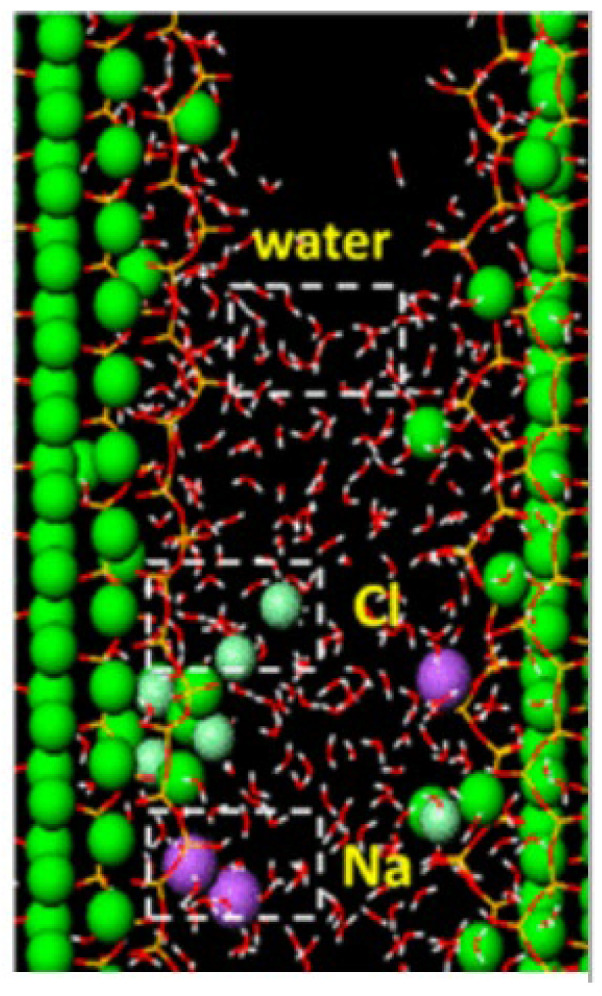
Transport model of Na^+^, Cl^−^ and water in the C-S-H slit-channel [67].

Hou DS et al. [67] used molecular dynamics to study the capillary transport of Na^+^, Cl^−^ and water in C-S-H nanochannel, as shown in Figure 9.

There is a strong correlation between surface calcium atoms, non-bridging oxygen, water and ions. The hydration structure of trapped ions and water in a C-S-H nanochannel has changed significantly. Moreover, these micropores provide conditions for the transmission of corrosive ions, leading to the deterioration of the internal components of concrete, and adversely affecting the service life and mechanical properties of concrete materials [68,69].

Inspired by the unique microstructure of nature, GO and C-S-H were reassembled into a layered structure with nano-scale gaps [70]. The confined space between the layers formed after recombination can be used as a 2D channel for the transmission of molecules and ions [71,72].

**Figure 10:**
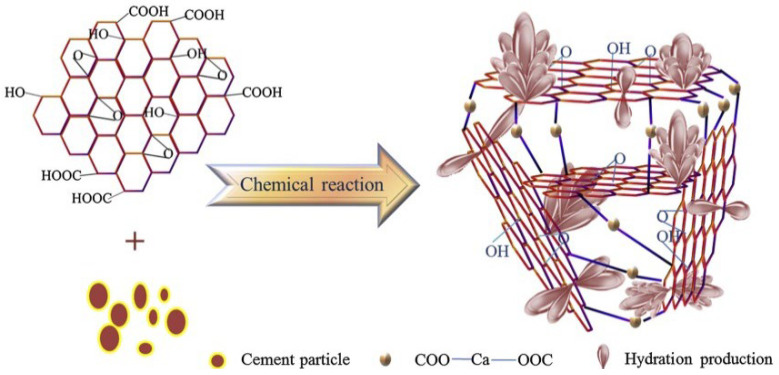
Interface structure model of hydration products (C-S-H) regulated by GO [73].

M. Wang et al. [73] proposed a 3D mechanism model of the GO/C-S-H interface by regulating with functional groups (see Figure 10). It is believed that -COOH at the edge of GO and Ca^2+^ of hydration product (Ca(OH)_2_) will form a 3D network structure of COO-Ca-OOC. At the same time, the hydration products are further inserted into the 3D nanostructure to compact the microstructure. As a result, the interface of cement-based composites can be regulated by GO [73]. D. S. Hou et al. [74] used experiments and molecular dynamics of the reaction field to study the interface structure and interface interaction mechanism between GO and C-S-H. It was found that the interfacial bonding between GO and C-S-H gel, and the instability of atoms in the interfacial region, are the reasons for the poor mechanical properties [74]. In addition, another work [75] used the molecular dynamics method to study water and ion transport in the nano-channels of the C-S-H matrix embedded with GO sheets. On the one hand, the transport rate and diffusivity of fluid largely depend on the types of functional groups in GO. Due to the invasion of ions and water molecules, the van der Waals interaction between graphene sheets and C-S-H gel is obviously weakened. On the other hand, the hydroxyl and carboxyl groups in the GO sheet provide enough oxygen sites to accept H bonds and combine with adjacent sodium ions, thus fixing water molecules and ions on the surface of GO. GO-COOH adsorbed on C-S-H further blocks the connectivity of the transport channel and “condenses” the water and ions in the entrance area of the gel hole [75].

1D carbon nanotubes and 2D nanostructures form nano-and sub-nano-scale ion channels with uniform size, and the internal microstructure and surface chemical characteristics of pores are more controllable. It can provide a reference for bionic multi-scale channels and other similar fields and has a clear leading role.

### 4.2. Application of GO Nano-Channels in Biomedical Engineering

Biological channels play an important role in life activities [76]. The study of the ion channel mechanism is of great significance to biophysics, bioinformatics and biomedicine. In order to realize the controllable transport of ions in GO nano-channels, the construction of artificial ion channels with various functions has become a research hotspot [77].

The biological channel is a pore-forming membrane protein. The biological channel plays an important role in complex life processes by controlling ion transport inside and outside the cell. However, natural channel proteins are extremely unstable. This limits its application as an in vitro experimental material. G. X. Li et al. [78] constructed a light-regulated ionic gate based on the design of a GO-biomimetic DNA-nano-channel architecture. Their research indicated that the single- and double-stranded DNA formed under the irradiation of alternating light has a different binding capacity with GO. Based on this, there are two conditions, that is, the adhesion and peeling of GO to the surface of the anodized aluminum film. It realizes the reversible conversion of ion gating between “off” and “on”. In addition, due to the high barrier property of GO and the extremely small diameter of the channel in the barrier layer, the ion gate constructed by them has an excellent switching efficiency and reversible ability of ion transport switch under alternating light irradiation. In addition, ion gating has an excellent switching efficiency and reversible ability of ion transport switch under alternating light irradiation. This can be attributed to the high barrier property of GO, and that the diameter of the channel in the barrier layer is extremely small [78].

The biological ion channel of GO also plays an important role in many physiological processes such as material transfer, energy conversion and signal transmission. Signals can be transmitted from nerve to brain in the process of sight, smell, hearing and touch based on biological ion channels. These functions are highly dependent on the high-speed ion transport of selective biological ion channels (10^7^ ions per second per channel) [79]. From the point of view of classical thermodynamics, the material transport of chemically selective nano-channels could be very slow. L. Jiang et al. [79] put forward the concept of “quantum confined superfluid” for the first time. They pointed out that the ordered superfluidity of ions and molecules in confined pores is a kind of “quantum tunneling fluid effect”. The “tunneling distance” is consistent with the period of quantum-confined superfluidity. In addition, the water in the biological channel is arranged in order by molecular chain. It indicates that the ultrafast transport of ions and molecules is carried out in a quantized way, namely “quantum confined superfluid”, such as the rapid transport of substances (10^6^ ions per second) in artificial ion channels and water channels [79].

Although a series of GO bionic nano-channels have been developed, a very complicated problem is to construct GO bionic nano-channels with stable, reversible, durable and fast transmission properties. From the experimental and theoretical point of view, it is still necessary to further study the transport mechanism of matter in GO bionic nano-channels, so as to make GO bionic nano-channels more controllable and intelligent.

### 4.3. Application of GO Nano-Channels in Environmental Engineering

Although three-quarters of the earth’s area is covered by water, there is very little water that can be safely drunk [80]. Even the Millennium Development Plan of the United Nations put the problem of “solving the shortage of drinking water” on the schedule. In recent years, the vigorous development of 2D materials has opened up a new direction of membrane separation for seawater desalination, and its advantages of high efficiency and low energy consumption have attracted wide attention [81].

71% of the earth is covered by water, but only 0.01% of the total water resources can be directly used by human beings [82]. According to statistics, about 663 million people in the world live in areas without a drinking water supply. It is estimated that the total population of the world will increase to 9 billion by 2050. At that time, mankind will encounter a huge crisis in the use of water resources, so it is urgent to solve the problem of freshwater shortage [83].

R. R. Nair and A. K. Geim [24] put forward the concept of molecular screening by GO film for the first time. They prepared sub-micron GO thin film by the spin coating method, and tested the permeability of various liquids and gases to GO thin film. It was found that the GO membrane showed a complete barrier to some organic solvents and gases (Ar, He, etc.), but almost had no obstacle to the permeation of water vapor. It can be attributed to the fact that hydrophilic FGs help attract water into the interlayer channels inside the GO membrane. At the same time, the friction-free super lubrication of water molecules in the channel enables water molecules to flow rapidly between GO layers [24]. The research proves that GO, a selective permeation characteristic, can be introduced into the field of seawater desalination. In 2014, R. K. Joshi et al. [25] explored the separation effect of the GO membrane on solutions containing different solutes through the experiment of solution permeation in U-shaped tubes separated by the GO membrane. It found that the shielding effect of GO film on solute in solution depends on the relationship between the size of the solute particles and the size of the GO channel [25]. After that, P. Z. Sun [84] discussed the permeation process of various salt ions in the GO membrane in more detail. They also confirmed that the GO membrane has a high screening effect on ions. For ions with different valence states, GO films will be separated at different degrees according to the charge amount [84]. These results further prove that GO has a special 2D channel, which can be used to selectively screen ions through the confinement effect of the channel. Therefore, it can be effectively used in seawater desalination by membrane separation.

## 5. Conclusions and Perspective

2D graphene-based nano-channels with controllable height at angstrom level were assembled by van der Waals force. The nano-channels have atomic smoothness and a highly clean surface, which can be used as a good platform for ion transport in confined 2D angstrom space [85]. The previous study [85] confirmed that graphene with angstrom-level (6.6–6.7 Å) nano-channels can completely intercept ions larger than 13 Å, while small-size ions can pass through the ion channels at different rates by squeezing or smoothing the hydration layer. With the increase in ion diameter, the energy to overcome also increases, so that ions with different hydration diameters have different transport rates. B. Chen et al. [86,87,88] used molecular dynamics to study the slip flow in interlayer channels of GO. It found that the wall slip velocity decreases with the increase in the oxidation degree, and the flow rate is linear with the slip velocity. Based on molecular motion theory and classical fluid mechanics, the essence of the slip flow in nano-channels is explained. The flow between nano-layers is attributed to the “positive effect” of slip flow and the “negative effect” of effective channel reduction [86,87,88]. It is of great significance to explore the special mass transfer phenomenon on the nano scale, and to reveal its physical mechanism and other basic scientific research.

With the development of micro/nano-manufacturing technology, nano or even sub-nano single channels with different geometries can be prepared [89]. Due to their special size effect and various interactions between ions or water molecules and the channel wall in the process of fluid transport, artificial nano-channels have shown excellent performance in many aspects, such as seawater desalination, water quality treatment, functional ion screening, energy storage and conversion, molecular sensing, etc. [90]. At present, there is a serious shortage of freshwater resources in the world, so it is imperative to use seawater desalination technology to produce fresh water [91]. The key technology is to use nano-channels to construct separation membranes to screen ions or desalinate brine. Among many nano-channels, low-dimensional carbon nano-channels, including 2D graphene nano-channels, one-dimensional carbon nanotube nano-channels and GO nano-channels, have become the best choice for preparing ion screening and seawater desalination membranes because of their ultra-high Young’s modulus, ultra-low interfacial friction resistance and outstanding ion and water molecule transport characteristics. In addition, studying the transport process of ions and water in nano-channels is of great significance for exploring the special mass transfer phenomena in nano scale and revealing its physical mechanism.

### 5.1. Capillary Condensation Effects

Capillary condensation refers to the phenomenon that the gas in the confined space of the capillary channel can condense and turn into liquid without supersaturation [92]. Capillary condensation, which is related to the wetting of macro-solid-liquid interface and micro-intermolecular mechanical action, is a key scientific problem of nano-confined mechanics, and it is also the international frontier hotspot of mesoscale science at present [93]. The Kelvin equation theoretically describes the change of vapor pressure caused by the curved liquid-gas interface in the capillary [94,95]. It is considered one of the three classical theories in the field of solid-liquid interface wetting. When the diameters of the nano-channels are reduced to the size of water molecules, the concepts of the meniscus curvature and contact angle used in the Kelvin equation are difficult to be accurately defined due to the complexity of experimental observation [96,97]. However, how to modify the Kelvin equation at the nanometer scale and how to cause capillary condensation in the confined space of the Angstrom scale are all problems worthy of further study.

### 5.2. Structural Design via Machine Learning

Machine learning (ML) is a branch of artificial intelligence. It uses computer algorithms to infer mathematical models, which can perform certain tasks directly from the acquired data, instead of based on established physical laws. The ML model can use a large amount of data to establish complex structure-property and composition-property relationships for desired compounds or generate new molecules and materials. In addition, it is also expected to improve the sensitivity and resolution of nano-channels detection by post-processing current data with novel analysis tools such as machine learning. Therefore, ML provides a powerful tool to accelerate the development of effective nano-channels, which can be used in various applications.

### 5.3. Folds and Geometric Effects

According to the different structures and chemical compositions of the nano-channels, the substances transported in the nano-channel are also different. External field (temperature, pH, light, etc.) and internal field (defects, heterojunctions, folds, etc.) will cause local deformation and initial stress of nano-channels, thus affecting physical properties such as mechanical properties and transport behavior of nano-channels.

For example, the environment (temperature, light, chemical composition) in which cells live is complex and changeable. These channels in the cell membrane must “learn” how to respond to the types and intensities of external stimuli, so that cells can survive in the complex environment.

The external field and internal field have certain regulatory functions on the transport and mechanical properties of nano-channels, and are different from those in 3-dimensional materials, so its physical mechanism is worth exploring.

### 5.4. Size Effects under Dynamics States

The dynamic size change of nano-channels is also very important. There will be uninterrupted curved nano-channels between cells during the physiological and pathophysiological processes. At present, artificial nano-channels mainly modify functional molecules on the inner surface of nano-channels by static methods to achieve a stimulation response. Therefore, ion transport regulation in static nano-channels is realized by adjusting their effective channel sizes. Hence, it is still a challenging task to give the artificial nano-channels dynamic shape changes and fixed channel sizes to control ion transport. The preparation methods of dynamic nano-channels can hardly give consideration to both flexibility and nano-size due to the selection of materials and the problem of nano-scale space blockage. Although adjustable elastomer nano-channels have been reported, the sizes of these nano-channels are more than three orders of magnitude of ion sizes, and they do not involve axial deformation.

## Data Availability

Not applicable.
